# The angiotensin II type 1 receptor antagonist telmisartan inhibits cell proliferation and tumor growth of esophageal adenocarcinoma via the AMPKa/mTOR pathway *in vitro* and *in vivo*

**DOI:** 10.18632/oncotarget.14345

**Published:** 2016-12-28

**Authors:** Shintaro Fujihara, Asahiro Morishita, Kana Ogawa, Tomoko Tadokoro, Taiga Chiyo, Kiyohito Kato, Hideki Kobara, Hirohito Mori, Hisakazu Iwama, Tsutomu Masaki

**Affiliations:** ^1^ Department of Gastroenterology and Neurology, Kagawa University Faculty of Medicine/Graduate School of Medicine, Kagawa 761-0793, Japan; ^2^ Life Science Research Center, Kagawa University, Kagawa 761-0793, Japan

**Keywords:** esophageal adenocarcinoma, telmisartan, AMPKα, angiotensin II type 1 receptor blocker, cell cycle arrest

## Abstract

Telmisartan, a widely used antihypertensive drug, is an angiotensin II type 1 (AT1) receptor blocker (ARB). This drug inhibits cancer cell proliferation, but the underlying mechanisms in various cancers, including esophageal cancer, remain unknown. The aim of the present study was to evaluate the effects of telmisartan on human esophageal cancer cell proliferation *in vitro* and *in vivo*. We assessed the effects of telmisartan on human esophageal adenocarcinoma (EAC) cells using the cell lines OE19, OE33, and SKGT-4. Telmisartan inhibited the proliferation of these three cell lines via blockade of the G_0_ to G_1_ cell cycle transition. This blockade was accompanied by a strong decrease in cyclin D1, cyclin E, and other cell cycle-related proteins. Notably, the AMP-activated protein kinase (AMPK) pathway, a fuel sensor signaling pathway, was enhanced by telmisartan. Compound C, which inhibits the two catalytic subunits of AMPK, enhanced the expression of cyclin E, leading to G_0_/G_1_ arrest in human EAC cells. In addition, telmisartan reduced the phosphorylation of epidermal growth factor receptor (p-EGFR) and ERBB2 *in vitro*. In our *in vivo* study, intraperitoneal injection of telmisartan led to a 73.2% reduction in tumor growth in mice bearing xenografts derived from OE19 cells. Furthermore, miRNA expression was significantly altered by telmisartan *in vitro* and *in vivo*. In conclusion, telmisartan suppressed human EAC cell proliferation and tumor growth by inducing cell cycle arrest via the AMPK/mTOR pathway.

## INTRODUCTION

Esophageal carcinoma is the eighth most common cancer worldwide and the sixth leading cause of cancer-related deaths [1]. It has one of the worst prognoses of any cancer, with a 5-year overall survival rate of approximately 15–25%. Diagnosis at advanced (metastatic) stages and metastasis are associated with poor prognosis [2, 3]. Esophageal adenocarcinoma (EAC) is less common than squamous cell carcinoma, but the frequency of adenocarcinoma of the esophagus, esophageal junction and gastric cardia has dramatically increased in Western countries [4]. EAC is among the most lethal cancers, with only 16% of patients surviving 5 years after diagnosis, and the median survival time is less than 1 year [5].

Telmisartan is an angiotensin II type 1 (AT1) receptor blocker (ARB) that is widely used as an antihypertensive drug. Several studies have indicated that angiotensin II promotes cell proliferation during cancer development, and ARBs suppress this effect by antagonizing the AT1 receptor [6–8]. ARBs inhibited the growth of breast [9], endometrial [10], and gastric cancer cells [11] in several *in vitro* and *in vivo* reports. Furthermore, epidemiological surveys have indicated that ARB treatment of hypertensive patients was associated with lower cancer incidence and mortality rates [12, 13].

Telmisartan is a partial agonist of peroxisome proliferator-activated receptor-gamma (PPAR-γ), activating the receptor to 25-30% of the maximum level achieved by the full agonist pioglitazone, a PPAR-γ ligand [14]. PPAR-γ activation inhibits cell growth in several cancers [15–18]. Additionally, telmisartan inhibits the proliferation of various cancer cell types, including prostate [19], renal [20] and colon [21] cancer cells, by inducing apoptosis.

AMP-activated protein kinase (AMPK) is a cellular energy sensor that is present in almost all eukaryotic cells [22]. It regulates cell growth and proliferation by modulating the mammalian target of rapamycin (mTOR) signaling pathway [23, 24]. AMPK is a possible therapeutic target for cancers with activated Akt signaling because AMPK inhibits mTOR, which is downstream of Akt [22]. More recently, telmisartan was shown to contribute to the activation of AMPK in vascular endothelial cells [25, 26]. However, little is known about the antitumor effect of telmisartan via AMPK/mTOR signaling in cancer cells.

Here, we demonstrate that telmisartan inhibited the growth of EAC cells by blocking cell cycle progression at the G_0_/G_1_ phase. Furthermore, telmisartan treatment activated the AMPK pathway and suppressed mTOR and p70S6 kinase (p70S6K) activation.

Thus, this study evaluated the effects of telmisartan on the growth of EAC cell lines and its mechanism of action. The miRNAs associated with the antitumor effect were also examined.

## RESULTS

### Telmisartan inhibits the proliferation and viability of human EAC cells *in vitro*

We examined the antitumor effects of telmisartan, irbesartan, losartan, and valsartan in three EAC cell lines *in vitro*. Cells were grown in 10% FBS and treated with 0, 1, 10, or 100 μM of the four ARBs (telmisartan, irbesartan, losartan, and valsartan) for 48 h.

**Figure 1:**
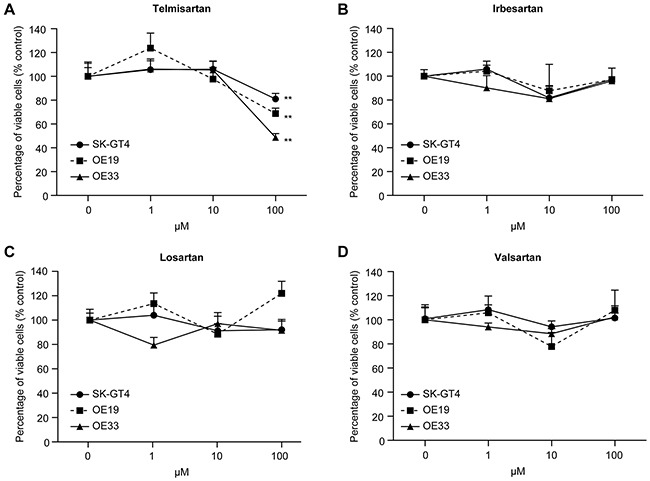
The effects of the ARBs telmisartan, irbesartan, losartan, and valsartan on the proliferation of EAC cell lines in vitro Telmisartan suppresses the proliferation of EAC cells. OE19, OE33, and SKGT-4 cells were seeded in 96-well plates. After 24 h, ARB (telmisartan, irbesartan, losartan, and valsartan; 1, 10, and 100 μM) or vehicle was added to the culture medium; 24 h later, the cells were evaluated with CCK-8 assays. Cell viability was assayed daily from 0 to 48 h. The viability of the ARB-treated cells was significantly different from that of the control cells (**, P < 0.01).

Telmisartan treatment (100 μM) reduced the proliferation of three EAC cell lines (OE19, OE33, and SKGT-4) (Figure 1A). None of the other ARBs (irbesartan, losartan, valsartan) affected the viability of the EAC cell lines (Figure 1B–1D). These results demonstrated that telmisartan strongly inhibits cell proliferation in the three EAC cell lines in a dose-dependent manner.

### Telmisartan induces cell cycle arrest in G_0_/G_1_ phase and regulates cell cycle-related proteins in EAC cells

**Figure 2:**
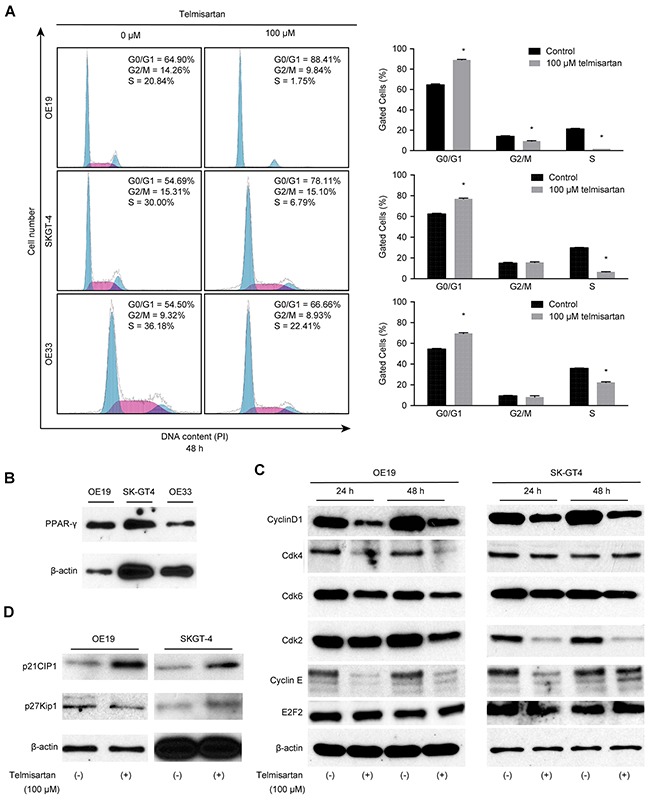
The antiproliferative effects of telmisartan in EAC cells are mediated via cell cycle arrest **A.** Cell cycle analysis of OE19, SKGT-4, and OE33 cells treated with 100 μM telmisartan at 48 h (*, P < 0.05). **B.** Western blot analysis of PPAR-γ in EAC cells. **C.** Western blot analysis of cyclin D1, Cdk4, Cdk6, Cdk2, cyclin E, and E2F2 in OE19 and SKGT-4 cells treated with 100 μM telmisartan. **D.** Western blot analysis of p21Cip1 and p27Kip in OE19 and SKGT-4 cells at 24 and 48 h after the addition of 100 μM telmisartan.

To further investigate the effects of telmisartan in the EAC cell lines (OE19, OE33, and SKGT-4), we examined cell cycle progression using flow cytometry analysis. Treatment with 100 μM of telmisartan increased the population of cells in G_1_ phase and reduced the populations of cells in the S and G_2_/M phases for 24–48 h after treatment (Figure 2A, Supplementary Figure 1).

Next, PPAR-γ expression was examined in EAC cell lines (OE19 and SKGT-4). The PPAR-γ protein level was significantly up-regulated in OE19 cells compared to that of SKGT-4 cells (Figure 2B). The effects of telmisartan on the expressions of various cell cycle-related proteins in OE19 and SKGT-4 cells were evaluated by western blotting. Cells were treated with 0 or 100 μM telmisartan for 24–48 h. The strongest reduction was observed in cyclin E and cyclin D1, key proteins involved in the transition from G_0_ to G_1_ phase, by telmisartan in a time-dependent manner (Figure 2C). In the present study, the telmisartan-mediated decrease in cyclin D1 and cyclin E in OE19 cells was only slightly greater than that in SKGT-4 cells. However, the PPAR-γ level in OE19 cells was significantly enhanced compared with that in SKGT-4 cells.

In addition, analysis of other proteins associated with the G_0_ to G_1_ transition indicated that Cdk4, the catalytic subunit of cyclin D1, was decreased in OE19 cells 24–48 h after the addition of telmisartan (Figure 2C). Cdk2, the catalytic subunit of cyclin E, was also decreased in SKGT-4 cells 24–48 h after the addition of telmisartan (Figure 2C). The protein levels of p21^Cip1^ also increased in response to telmisartan treatment (Figure 2D). PPAR-γ ligands were previously reported to inhibit the mRNA expressions of cyclin E and E2F2 in a colon cancer cell line [16]. However, E2F2 expression in EAC cells was not detected following treatment with telmisartan (Figure 2C).

These findings suggest that telmisartan inhibits cell cycle progression from G_0_/G_1_ to S-phase by decreasing cyclin D1 and cyclin E levels, which results in G_1_ cell cycle arrest, in a PPAR-γ-independent manner in EAC cells. We therefore focused on the major pathway of telmisartan-induced cell cycle arrest in our next experiments.

### Telmisartan induces the phosphorylation of AMPK and regulates cell cycle-related proteins via the AMPK/mTOR pathway in EAC cells

**Figure 3:**
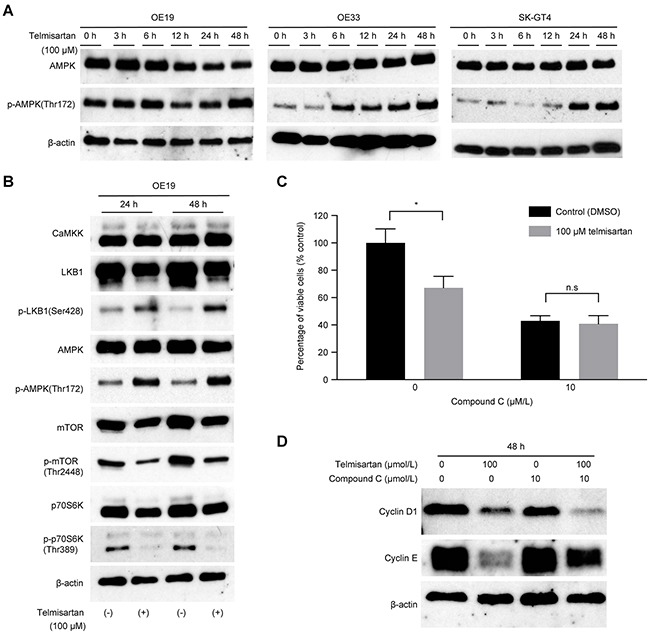
Telmisartan induces cell cycle arrest via activation of the AMPK pathway and suppression of mTOR signaling **A.** Three EAC cell lines were treated with 100 μM telmisartan for the indicated times, and the activation status of AMPKα was assessed by western blotting. **B.** OE19 cells were treated with 100 μM telmisartan, and the activation status of the LKB1/AMPK/mTOR pathway was determined. **C.** The antiproliferative effects of telmisartan or the control in combination with various concentrations of compound C were assessed in OE19 cells for 48 h. **D.** Western blot analysis of cyclin D1 and cyclin E in OE19 cells treated with control, telmisartan alone, compound C alone, or telmisartan combined with compound C for 48 h. n.s., not significant; *, P<0.05.

To evaluate the mechanism of telmisartan-induced cell cycle arrest, we focused on AMPK/ mTOR signaling. Telmisartan induced the phosphorylation of AMPKα at Thr^172^ in three EAC cell lines, and this activation lasted for at least 48 h (Figure 3A). The protein levels of p-mTOR and p-p70S6K decreased in OE19 cells following telmisartan treatment (Figure 3B). Telmisartan also increased the phosphorylation of LKB1. However, no significant changes were observed in CaMKK expression (Figure 3A).

To determine whether the antiproliferative effect of telmisartan is mediated by AMPKα/mTOR signaling, we blocked this pathway using compound C, which targets the two catalytic units of AMPK. The antiproliferative effect of telmisartan was attenuated by compound C compared with that of the control (Figure 3C). More importantly, telmisartan induced antiproliferative effects via the AMPKα/mTOR pathway by inhibiting cell cycle regulatory molecules, especially cyclin E (Figure 3D).

These data indicate that telmisartan regulates cell cycle-related protein levels by phosphorylation of AMPKα at Thr^172^ and induces antiproliferative effects in EAC cells.

### Telmisartan inhibits the activation of RTKs, downstream effectors and cell cycle-related proteins

**Figure 4:**
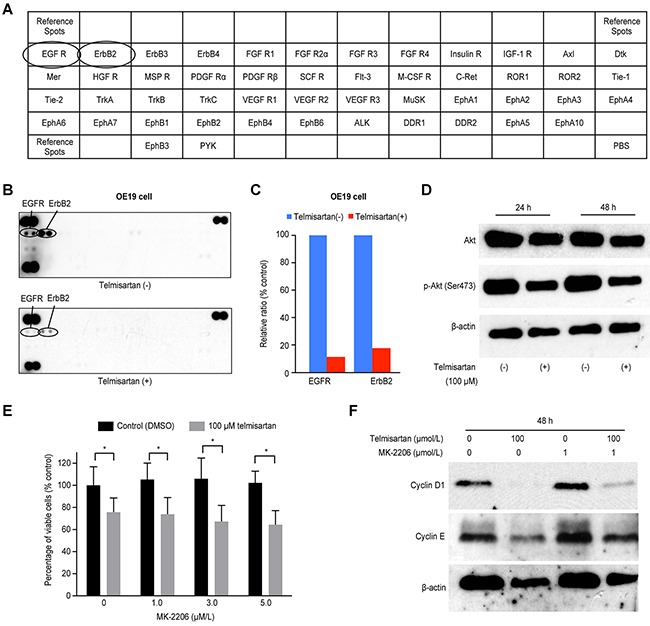
**A.** The template indicates the locations of tyrosine kinase antibodies spotted onto a human phospho-RTK array. **B.** Representative expression of various phosphorylated tyrosine kinase receptors in OE19 cells treated with or without 100 μM telmisartan at 24 h. **C.** Densitometry indicated that the ratios of p-EGFR and ERBB2 spots of telmisartan-treated to untreated cells were 11.6% and 17.5%, respectively. **D.** Western blot analysis of Akt and p-Akt (Ser473), which are downstream of EGFR signaling, in EAC cells treated with 100 μM telmisartan. **E.** The antiproliferative effects of telmisartan or the control in combination with various concentrations of MK-2206 were assessed in OE19 cells for 48 h. (D) Western blot analysis of cyclin D1 and cyclin E in OE19 cells treated with the control, telmisartan alone, MK-2206 alone, or telmisartan combined with MK-2206 for 48 h. *,P<0.05.

We used a p-RTK array to identify the key RTKs associated with the antitumor effects of telmisartan. Using an antibody array (Figure 4A), we simultaneously analyzed the expressions of 46 different activated RTKs in OE19 cells 24 h after telmisartan administration. Telmisartan reduced the expression of phosphorylated epidermal growth factor receptor (p-EGFR) and ERBB2 *in vitro* (Figure 4B). The densitometric analyses of p-EGFR and p-ERBB2 showed decreases of 11.6% and 17.5%, respectively (Figure 4C). In addition, we evaluated the protein levels of Akt and p-Akt, which are downstream of EGFR. Telmisartan decreased the expression of both Akt and p-Akt (Figure 4D).

Furthermore, to determine whether the antiproliferative effects of telmisartan were mediated via the Akt pathway, we tested the Akt inhibitor MK-2206 in OE19 cells (Figure 4E). The expressions of cyclin D1 and cyclin E were reduced by telmisartan, and this effect was slightly attenuated by MK-2206 (Figure 4F). Thus, telmisartan may partially inhibit cell cycle regulatory molecules through the Akt/mTOR signaling pathway to control cell proliferation in EAC cells.

### Telmisartan inhibits tumor proliferation *in vivo*

**Figure 5:**
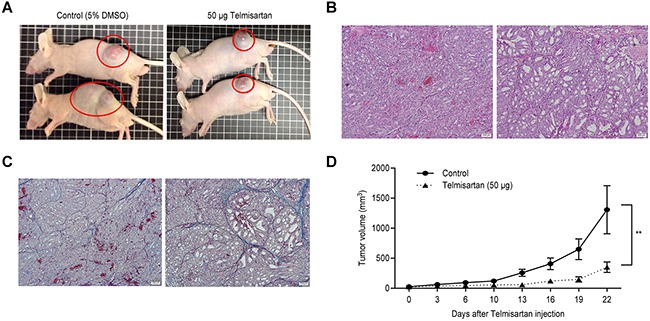
The growth of OE19-derived tumors in mice treated with telmisartan is inhibited **A.** Representative images of gross OE19 tumors from nude mice treated with vehicle (i) or 50 μg of telmisartan. (ii). **B.** Histological examination using H and E staining. **C.** Fibrotic tissue as determined by Azan staining of subcutaneous xenograft tumors 22 days after telmisartan injection. **D.** Tumor growth curves of the control and telmisartan groups. Tumor volume (mm3) was calculated as (tumor length (mm) × tumor width (mm)2)/2. The tumors were significantly smaller in the telmisartan-treated mice than those in the vehicle-treated mice. Each point represents the mean ± standard deviation of 7 animals. P = 0.0007 by two-way ANOVA.

To determine whether telmisartan could affect tumor growth *in vivo*, we subcutaneously injected nude mice with OE19 cells, followed by i.p. injection of telmisartan. The telmisartan treatment inhibited tumor growth by 73.2%, as determined by integrated tumor growth curves (Figure 5A and 5D), compared with that of the untreated control mice. Fibrotic areas in the tumors derived from OE19 cells were unaffected by telmisartan (Figure 5B and 5C). Telmisartan had no apparent toxic effects on the mice and no effect on body weight during the study. Furthermore, all animals survived to the end of the experiment.

### Telmisartan affects miRNA expression

Using a custom microarray platform, we analyzed the expression levels of 2555 miRNA probes in cell lines and tumor tissues in the presence and absence of telmisartan (GEO, accession no. GSE81350, GSE81354). Treatment with 100 μM telmisartan for 48 h up-regulated 10 miRNAs and down-regulated 15 miRNAs in OE19 cells (Supplementary Table 1). In the tumor xenograft model, there were 2 up-regulated and 6 down-regulated miRNAs in the telmisartan group as determined using the custom microarray platform (Supplementary Table 2).

Unsupervised hierarchical clustering analysis was conducted using Pearson's correlation, and the results indicated that cell lines *in vitro* and tumor tissues *in vivo* treated with telmisartan clustered together and separately from untreated cell lines and tissues (Supplementary Figure 4).

## DISCUSSION

The ARB telmisartan is one of the most commonly prescribed antihypertensive drugs. Telmisartan has been shown to block cancer cell proliferation [6–8] *in vitro* and tumor growth *in vivo* [9–11]. Recently, a retrospective study found that treatment with ARBs and angiotensin-converting enzyme inhibitors is not associated with survival in esophageal cancer [27]. However, the antitumor effects of telmisartan in EAC remained unknown. We demonstrate here for the first time that telmisartan has antitumor effects in EAC *in vitro* and *in vivo*. Telmisartan induced cell cycle arrest at the G_0_/G_1_ phase by modulating the expression of cell cycle regulatory proteins in EAC cells.

Our flow cytometric analyses demonstrated that telmisartan significantly induced cell cycle arrest in EAC cells. These findings were further corroborated by an analysis of cell cycle-related proteins. A substantial reduction was observed in the cell cycle regulatory proteins cyclin E, cyclin D1, and CDKs. Specific cyclin/Cdk complexes are activated at different times during cell cycle progression. Complexes of Cdk4 and Cdk6 with cyclin D1 are required for G_1_ phase progression, whereas complexes of Cdk2 with cyclin E are required for the G_1_-S transition [28]. The expression of various cell cycle-related molecules has been shown to enhance esophageal cancer and is related to cancer prognosis [29, 30]. These data indicate that the major cell cycle regulators (cyclin D1 and cyclin E) may be intracellular targets of telmisartan in human EAC cell lines. Therefore, inhibition of these molecules, including cyclin E and cycle D1, may be a promising strategy for controlling human esophageal cancer.

Telmisartan has been shown to activate AMPK in vascular endothelial cells [25, 26] but not in cancerous cells. Kurokawa et al. reported that telmisartan independently enhanced the AMPK signaling pathway [25]. In the present study, telmisartan activated the AMPK pathway and inhibited p70S6K and mTOR phosphorylation in EAC cells. In addition, the telmisartan-mediated inhibition of cyclin E was attenuated by an AMPKα inhibitor. These data indicate that telmisartan induces antiproliferative effects by phosphorylation of AMPKα at Thr^172^ in EAC cells, suggesting that the activation of the AMPKα/mTOR pathway inhibits cell cycle regulatory molecules. AMPK activation has recently been shown to inhibit the mTOR pathway and p70S6K phosphorylation, which are involved in protein synthesis. The results suggest that this pathway may regulate cell proliferation in various cancer cells [31–33]. These reports support our finding that AMPKα/mTOR signaling is a pivotal pathway in the telmisartan-induced antiproliferative effects.

**Figure 6:**
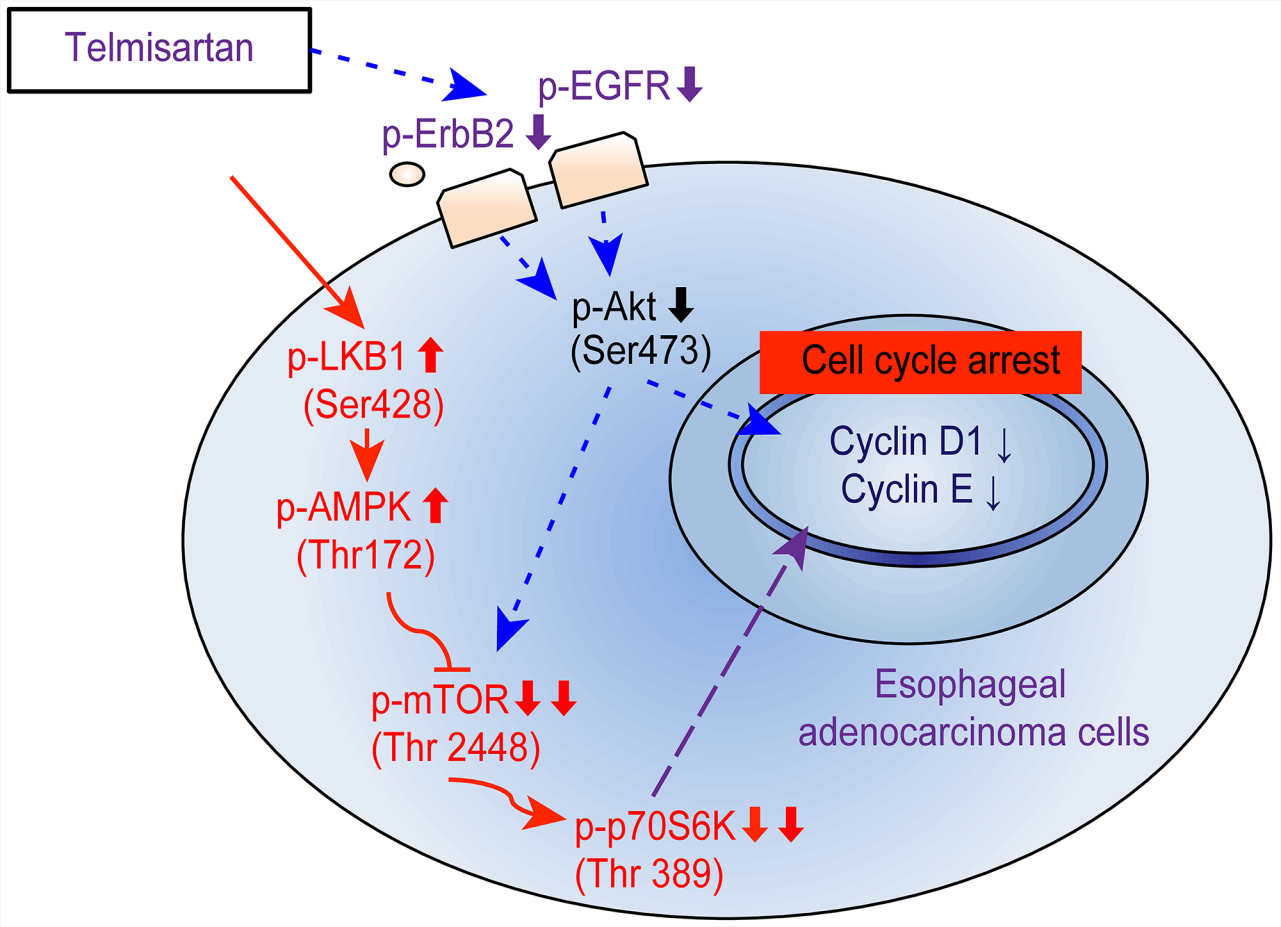
Schematic model for telmisartan inhibition of cell proliferation and G1 cell cycle progression in EAC cells The activation of AMPKα in human EAC cells inhibits mTOR and p70S6K, inducing G1 cell cycle arrest. Telmisartan may affect the cell cycle via the phosphorylation of EGFR and Akt/mTOR.

Activating AMPKα also suppressed cancer cell proliferation via increased expression of the cell cycle inhibitor p21 [34, 35]. Additionally, AMPKα has been linked to two tumor suppressors, LKB1 and CaMKK. LKB1 is an upstream kinase that phosphorylates and activates AMPKα in physiological settings [34]. Telmisartan activated LKB1 and AMPKα without affecting CaMKK and suppressed mTOR activity in EAC cells. These results indicate that telmisartan reduced the expression of cell cycle-related proteins and induced cell cycle arrest via the AMPKα/mTOR pathway in EAC cells (Figure 6).

Telmisartan is an ARB; thus, AT1R may be involved in telmisartan-induced cell cycle arrest in EAC. AT1R induces cell proliferation in multiple cancer cells by activating various intracellular protein kinase cascades associated with growth factor stimulation [36]. We found that telmisartan inhibited EAC cell proliferation, but the ARBs irbesartan, valsartan, and losartan did not have antiproliferative effects in the three EAC cell lines. AT1R was expressed in several EAC cell lines (Supplementary Figure 5A). Notably, AT1R transactivates EGFR in several cancers [37], leading to extracellular-regulated kinase (ERK) activation, phosphorylation of signal transducer and activator of transcription 3 (STAT3) and activation of protein kinase C (PKC). Among these pathways, MEK/ERK can regulate cell cycle progression, apoptosis or differentiation. Therefore, we focused on MEK/ERK signaling to elucidate the mechanism of telmisartan-induced cell cycle arrest. The protein levels of p-p42/44 (Erk1/2) decreased in OE19 cells following telmisartan treatment, and these effects lasted for at least 48 h (Supplementary Figure 5B). Furthermore, to determine the antiproliferative effects of telmisartan via the MEK/ERK pathway, we tested the ERK1/2 inhibitor SCH772984 in OE19 cells. SCH772984 is a novel and selective inhibitor of ERK1/2 that displays characteristics of both type I and II kinase inhibitors [38]. The expressions of cyclin D1 and cyclin E were not changed by telmisartan, and this effect was not attenuated by SCH772984 (Supplementary Figure 5C). In addition, SCH772984 did not affect telmisartan-induced down-regulation of cyclin D1 and cyclin E, even though the phosphorylation of Erk1/2 were obviously reduced by telmisartan. Therefore, telmisartan did not induce cell cycle arrest by decreasing the cell cycle regulatory molecules through the MEK/ERK signaling pathway, and it is independent of the AT1R cascade in EAC cells.

Telmisartan is a partial agonist of PPAR-γ and influences the expression of PPAR-γ target genes involved in carbohydrate and lipid metabolism [14]. Telmisartan is the strongest PPAR-γ partial agonist among the ARBs (irbesartan, candesartan, valsartan) [14]. Other PPAR-γ partial agonists also inhibit cancer cell proliferation by inducing cell cycle arrest [16–18]. Troglitazone, which is a strong activator of PPAR-γ, was previously reported to inhibit the mRNA levels of cyclin E and E2F2 in a colon cancer cell line [16]. In this study, no significant reduction in E2F2 was identified following telmisartan treatment, although telmisartan significantly decreased cyclin E expression. The antiproliferative effects of PPAR-γ induced by telmisartan treatment were lower than those induced by troglitazone treatment because telmisartan is a partial agonist of PPAR-γ [14, 15]. In addition, the telmisartan-mediated decrease in cyclin D1 and cyclin E was similar in OE19 and SKGT-4 cells despite the large difference in PPAR-γ expression between these cell lines. Therefore, these results suggest that PPAR-γ does not play a major role in the inhibition of EAC cell proliferation and tumor growth caused by telmisartan-induced cell cycle arrest.

Additionally, we demonstrated that telmisartan reduces the phosphorylation of EGFR and ERBB2 in EAC cells using p-RTK arrays. EGFR activation induced the expression of cyclin D1, a key protein in cell cycle progression [31]. In this study, telmisartan decreased the expression level of Akt. mTOR, a major downstream target of Akt, regulates p70S6K. mTOR and p70S6K are downstream of PI3K and Akt in pathways regulating G_1_ cell cycle progression [39]. We examined the antiproliferative effects of telmisartan mediated by Akt using MK-2206 in OE19 cells. The expressions of cyclin D1 and cyclin E were reduced by telmisartan, and these reductions were slightly attenuated by MK-2206. A previous study showed that PPAR-γ partial agonists induce cell cycle arrest by reducing the levels of p-EGFR, Akt, and p21 in esophageal squamous cell carcinoma [17]. Thus, telmisartan may partially inhibit cell cycle regulatory proteins via the Akt/mTOR pathway to control cell proliferation in EAC cells.

Telmisartan has been shown to inhibit cell proliferation by inducing apoptosis in various cancer cell lines, including endometrial [10], prostate [19], renal [20], and colon [21] cancer lines. To determine whether telmisartan induced apoptosis, we treated EAC cells with or without 100 μM telmisartan for 24 and 48 h and analyzed the cells using flow cytometry. Telmisartan did not increase the proportion of apoptotic cancer cells 48 h after treatment in OE19, SKGT-4, and OE33 cells (Supplementary Figures 2-3). These results indicate that telmisartan inhibits EAC cell proliferation without inducing apoptosis.

Telmisartan also markedly suppressed the growth of subcutaneous EAC tumors in athymic nude mice. Our *in vitro* study was conducted using a higher dose of telmisartan than that used in human treatments (1–10 μM) [14, 40, 41]. However, the use of high doses has been criticized in similar studies examining other cancer cell types, such as breast [9], stomach [11], and prostate cancer cells [19]. Our *in vivo* study was conducted using a slightly higher dose of telmisartan than that used in human administration.

Recently, candesartan, another ARB, was shown to significantly reduce transforming growth factor β1 (TGF-β1) expression and suppress tumor proliferation and stromal fibrosis [11]. Candesartan also significantly inhibited the growth of tumor xenografts and angiogenesis in mice [11]. Telmisartan reduced VEGF, TIMP-1, and angiogenin levels in OE19 cells (Supplementary Figure 3). However, fibrotic areas in the implanted tumor derived from OE19 cells were not decreased by telmisartan in the xenograft models. This result is anomalous—reduced expression of TIMP-1 did not affect the activation of MMPs, which degrade collagen deposition. One explanation for this result could be the difference between *in vitro* and *in vivo* effects. In an *in vivo* mouse model, non-cancerous cells, such as fibroblasts, endothelial cells, and inflammatory cells, that are adjacent to cancer cells may affect the development of esophageal cancer via cell-cell interactions in the solid tumor microenvironment.

MicroRNAs, small non-coding RNA molecules, regulate the development and progression of various cancers [42]. Several microRNAs were significantly altered following telmisartan treatment *in vitro* and *in vivo*. Among these microRNAs, miR-200a and miR-301a were significantly down-regulated in OE19 cells treated with telmisartan. To clarify the relationship between p-AMPKα and these miRNAs, we further assayed the effect of miR-201a-3p and miR-301a-3p overexpression on the expression and phosphorylation of AMPKα in OE19 cells treated with or without telmisartan. miR-200a-3p overexpression did not attenuate p-AMPKα regardless of telmisartan treatment (Supplementary Figure 6). However, the expression of p-AMPKα was induced by telmisartan, and this effect was slightly attenuated by miR-301a-3p (Supplementary Figure 6). Thus, miR-301a-3p may regulate the phosphorylation of AMPKα through the AMPKα/mTOR signaling pathway to control cell proliferation in EAC cells.

Several reports have already shown that i) miR-200a directly regulates A2, a receptor for the oncogene Eph, and decreases cancer cell migration via downstream activation of AMPKα [43], and ii) miR-301a appears to directly down-regulate AMPKα1 in osteosarcoma cells [44]. These findings support our results indicating that telmisartan inhibited cancer proliferation and tumor growth via AMPK activation, which was likely enhanced by the down-regulation of miR-200a and miR-301a. In addition, there were no matched miRNAs extracted from cultured cells and implanted tumor tissues in the present study (Supplementary Figure 4). This also indicates that cell-cell interactions between tumor cells and stromal cells may alter the tumor microenvironment.

In conclusion, our results revealed that telmisartan inhibits human EAC cell proliferation and tumor growth, inducing cell cycle arrest by regulating cell cycle-related molecules via the AMPK/mTOR pathway.

## MATERIALS AND METHODS

### Chemicals

Telmisartan and valsartan were purchased from Tokyo Chemical Industry Co. (Tokyo, Japan). Irbesartan and losartan were purchased from Wako Pure Chemical Industries (Osaka, Japan). Telmisartan was prepared as a 10 mM stock solution in dimethyl sulfoxide (DMSO). Irbesartan and valsartan were prepared as 100 mM stock solutions in DMSO, and losartan was prepared as a 100 mM stock solution in H_2_O. The stock solutions were stored at -20°C. Compound C was purchased from Abcam (Cambridge, UK). MK-2206 was purchased from ChemScene Chemicals (Monmouth Junction, NJ, USA). SCH772984 was purchased from Selleck Chemicals (Houston, TX, USA).

### Cell culture and cell proliferation assay

OE19, OE33, and SKGT-4 human EAC cell lines were obtained from the European Collection of Authenticated Cell Cultures (ECACC). All cell lines were grown in RPMI-1640 (Gibco Invitrogen, Carlsbad, CA) supplemented with 10% fetal bovine serum (FBS) and penicillin-streptomycin (100 mg/L; Invitrogen) at 37°C in a humidified atmosphere containing 5% CO_2_.

Cell proliferation was assayed using the CCK-8 cell counting kit according to the manufacturer's instructions. Briefly, 5 × 10^3^ cells were seeded into each well of a 96-well plate and cultured in 100 μL of RPMI-1640 supplemented with 10% FBS. After 24 h, ARBs (telmisartan, irbesartan, losartan, and valsartan at 0, 1, 10, or 100 μM) or vehicle was added to each well, and cells were cultured for an additional 48 h. CCK-8 reagent (10 μL) was added to each well, and the plates were incubated at 37°C for 3 h. The absorbance was measured at 450 nm using a microplate reader.

### Preparation of cell lysates

Cell lysates were prepared as previously described at 4°C [45]. Protein concentrations were measured using a dye-binding protein assay based on the Bradford method [46].

### Gel electrophoresis and western blotting

OE19 cells (1.0 × 10^6^/dish) were seeded in 100 mm culture dishes and cultured for 24 h. Then, 100 μM telmisartan was added, and the cells were further cultured for 24-48 h. The cells were lysed in a protease inhibitor cocktail (“complete” protease inhibitor mixture; iNtRON Biotechnology, Seongnam, Korea) on ice for 20 min. Cell lysates were centrifuged at 13,000 × g at 4°C for 5 min. Supernatants containing the soluble cellular proteins were collected and stored at −80°C until use. Protein concentrations were measured using a NanoDrop 2000 fluorospectrometer (Thermo Scientific Corporation, USA). Protein aliquots (1–10 μg) were resuspended in sample buffer and separated on 10% Tris-glycine gradient gels via SDS-PAGE [47]. The proteins were subsequently transferred to nitrocellulose membranes. The membranes were blocked and then incubated with primary antibodies followed by horseradish peroxidase (HRP)-conjugated secondary antibodies [48].

Primary antibodies used for western blot analyses were obtained from the following sources: β-actin antibody was obtained from Sigma-Aldrich (St. Louis, MO, USA); cyclin D1 and cyclin E antibodies were obtained from Thermo Fisher Scientific (Waltham, MA, USA); Cdk6, Cdk2, and Cdk4 antibodies were obtained from Santa Cruz Biotechnology (Santa Cruz, CA, USA); phosphorylated retinoblastoma protein and p27^Kip1^ antibodies were obtained from BD Biosciences Pharmingen (San Jose, CA, USA); PPAR-γ (ab19481), E2F2 (ab65222), and AT1 receptor (ab9391) antibodies were purchased from Abcam (Abcam, USA); AMPKα (#5832), p-AMPKα Thr172 (#2535), LKB1 (#3047), p-LKB1 Ser428 (#3482), CaMKK (#4436), mTOR (#2983), p-mTOR Ser2448 (#5536), p70S6K (#2708), p-p70S6K Thr389 (#9205), Akt (#4685), p-Akt Ser473 (#4060), p21^Waf1/Cip1^ (#2947), p-Erk1/2 (#4370), and Erk1/2 (#4695) antibodies were purchased from Cell Signaling Technology (Boston, MA, USA).

HRP-linked anti-mouse and anti-rabbit IgG secondary antibodies (1:2000; Cell Signaling Technology, MA, USA) were used.

Immunoreactive proteins were visualized with an enhanced chemiluminescence detection system (PerkinElmer Co., Waltham, MA, USA) on X-ray film.

### Cell cycle and apoptosis analysis

Cell cycle profiles were analyzed after telmisartan treatment to assess growth inhibition. OE19, OE33, and SKGT-4 cells (1.0 × 10^6^ cells in a 100 mm diameter dish) were treated with or without 100 μM telmisartan for 24–48 h. Cell cycle progression was analyzed by measuring the amount of propidium iodide (PI)-labeled DNA in ethanol-fixed cells. The fixed cells were washed with PBS and then stored at −20°C for flow cytometry analysis. On the day of analysis, the cells were washed with cold PBS, suspended in 100 μL of PBS with 10 μL of RNase A (250 μg/mL) and incubated for 30 min. A 110 μL aliquot of PI (100 μg/mL) was added to each suspension, and the cells were incubated at 4°C for at least 30 min prior to analysis. Apoptotic and necrotic cell death was analyzed by double staining with FITC-conjugated Annexin V and PI, which is based on the binding of Annexin V to apoptotic cells with exposed phosphatidylserine and PI labeling of late apoptotic/necrotic cells with membrane damage. Tumor cells were treated for 24 and 48 h. Staining was performed according to the manufacturer's instructions. Flow cytometry was performed using a Cytomics FC 500 flow cytometer (Beckman Coulter, Indianapolis, IN, USA). Cell percentages were determined using Kaluza software (Beckman Coulter, Indianapolis, IN, USA). All experiments were performed in triplicate.

### Antibody arrays of phosphorylated receptor tyrosine kinases (p-RTKs)

Human p-RTKs were assayed using Human Phospho-RTK Array Kits (R&D Systems, Minneapolis, MN, USA) according to the manufacturer's instructions. Briefly, p-RTK array membranes were blocked with 5% BSA/TBS (0.01 M Tris-HCl, pH 7.6) for 1 h and incubated with 2 mL of lysate, which was prepared from cell lines after normalization to ensure equal protein amounts. The membranes were washed 3 times with TBS plus 0.1% v/v Tween-20 for 10 min each and 2 times with TBS alone for 10 min each to remove unbound materials. Then, the membranes were incubated with an HRP-conjugated anti-phospho-tyrosine antibody for 2 h at room temperature. The unbound HRP-conjugated antibody was washed away with TBS plus 0.1% Tween-20. Finally, each array membrane was exposed to X-ray film using a chemiluminescence detection system (PerkinElmer Co.). The immunoreactive bands were analyzed by densitometric scanning (TIc scanner, Shimizu Co., Ltd., Kyoto, Japan).

### Angiogenic profile analysis using an antibody array

A Human Angiogenesis Antibody Array (R&D Systems, Minneapolis, MN, USA) was used according to the manufacturer's protocol. This method is a dot-based assay enabling the detection and comparison of 55 angiogenesis-specific cytokines. Each array membrane was exposed to X-ray film using a chemiluminescence detection system (PerkinElmer Co.).

### Xenograft model analysis

Animal experiments were performed according to the guidelines of the Committee on Experimental Animals of Kagawa University, Kagawa, Japan.

Male athymic mice (BALB/c-nu/nu; 6 weeks old; 20–25 g) were purchased from Japan SLC, Inc. and maintained under specific pathogen-free conditions using a laminar airflow rack. The mice had continuous free access to sterilized (γ-irradiated) food (CL-2; CLEA Japan, Inc.) and autoclaved water. Each mouse was subcutaneously inoculated with OE19 cells (5 × 10^6^ cells per animal) in the flank. One week later, the xenografts were identifiable as masses with a maximal diameter > 4 mm. The animals were randomly assigned to treatment with telmisartan (50 μg per day) or diluent only (control). The telmisartan group was intraperitoneally (i.p.) injected five times per week with 2 mg/kg telmisartan for four weeks; the control group was administered 5% DMSO alone for four weeks. Tumor growth was monitored daily by the same investigators (S. Fujihara and A. Morishita), and tumor size was measured weekly. The tumor volume (mm^3^) was calculated as the tumor length (mm) × tumor width (mm)^2^/2 [49]. All animals were sacrificed on day 22 after treatment, and all animals survived during this period. Between-group differences in tumor growth were analyzed by two-way ANOVA.

### miRNA arrays

Total RNA was extracted from the tumor samples and cancer cell lines using a miRNeasy Mini Kit (Qiagen, Hilden, Germany) according to the manufacturer's instructions. RNA samples typically exhibited A_260/280_ ratios between 1.9 and 2.1, as determined using an Agilent 2100 Bioanalyzer (Agilent Technologies, Santa Clara, CA, USA).

After RNA measurements were performed with an RNA 6000 Nano Kit (Agilent Technologies), the samples were labeled using a miRCURYHy3/Hy5 Power Labeling Kit and were subsequently hybridized to a human miRNA Oligo chip (v. 21.0; Toray Industries, Tokyo, Japan). The chips were scanned with a 3D-Gene Scanner 3000 (Toray Industries), and the results were analyzed using 3D-Gene extraction version 1.2 software (Toray Industries). Differences in miRNA expression between the telmisartan-treated and control samples were assessed using GeneSpring GX v10.0 (Agilent Technologies). Quantile normalization was performed on the raw data that were above the background level. Differentially expressed miRNAs were determined by the Mann-Whitney U test. The false discovery rate was computed with the Benjamini-Hochberg method [50] for multiple testing. Hierarchical clustering was performed using the furthest neighbor method with the absolute uncentered Pearson's correlation coefficient as a metric. A heat map was produced with the relative expression intensity for each miRNA, in which the base-2 logarithm of the intensity was median-centered for each row.

### Gene transfection

miR-200a-3p mimics, miR-301a-3p mimics, and negative control miRNA were obtained from Thermo Scientific (Waltham, MA, USA). OE19 cells were seeded in 6-well plates. After 24 h, OE19 cells were transfected with miR-200a-3p mimic, miR-301a-30 mimic, or negative control miRNA at a final concentration of 10 nM using Lipofectamine RNAiMAX (Invitrogen, Grand Island, NY, USA). After a 24 h incubation, the cells were harvested and washed with ice-cold PBS for subsequent analysis.

### Statistical analyses

All statistical analyses were performed using Prism 6 software (GraphPad Software, La Jolla, CA, USA). Comparisons between treatment and control groups were performed using two-tailed paired or unpaired Student's *t* tests. A *P*-value of < 0.05 was considered significant.

## SUPPLEMENTARY MATERIALS FIGURES AND TABLES



## Abbreviations

EAC: esophageal adenocarcinoma
ARBs: angiotensin II type 1 receptor blockers
AT1: angiotensin II type 1
PPAR-γ: peroxisome proliferator-activated receptor-gamma
AMPK: AMP-activated protein kinase
EGFR: epidermal growth factor receptor
DMSO: dimethyl sulfoxide
CCK-8: cell counting kit
