# Supplementary material for: The angiotensin II type 1 receptor antagonist telmisartan inhibits cell proliferation and tumor growth of esophageal adenocarcinoma via the AMPKa/mTOR pathway in vitro and in vivo
Source: Oncotarget. 2016 Dec 28;8(5):8536–49. doi: 10.18632/oncotarget.14345 (PMC5352420; doi:10.18632/oncotarget.14345)
Supplement: Supplementary file 1 [file oncotarget-08-8536-s001.pdf]

The angiotensin II type 1 receptor antagonist telmisartan inhibits cell proliferation and tumor growth of esophageal adenocarcinoma via the AMPKα/mTOR pathway *in vitro* and *in vivo*

SUPPLEMENTARY FIGURES AND TABLES

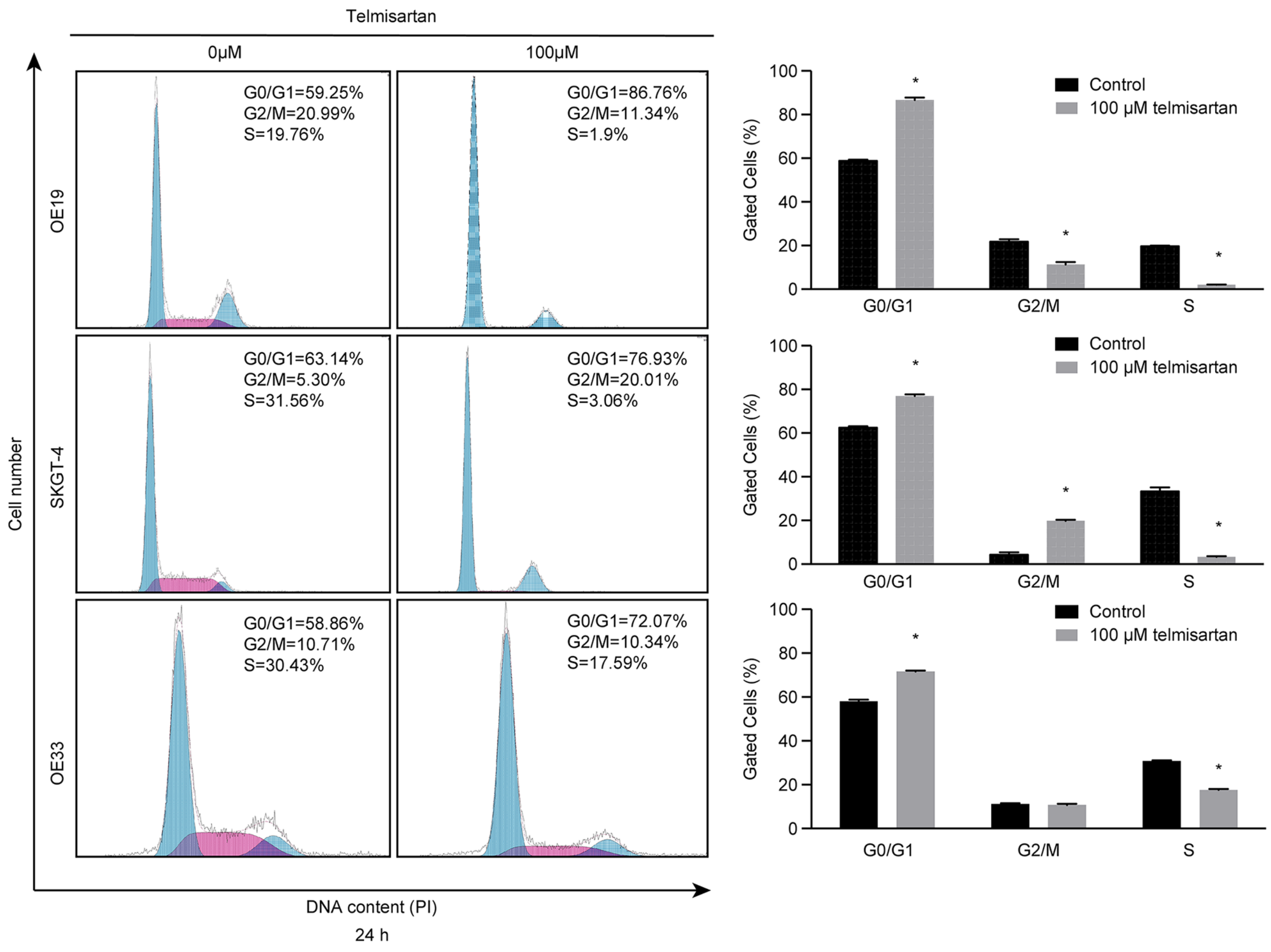

Supplementary Figure S1: Cell cycle analysis of OE19, SKGT-4, and OE33 cells treated with 100 μM telmisartan at 48 h. *, *P*<0.05.

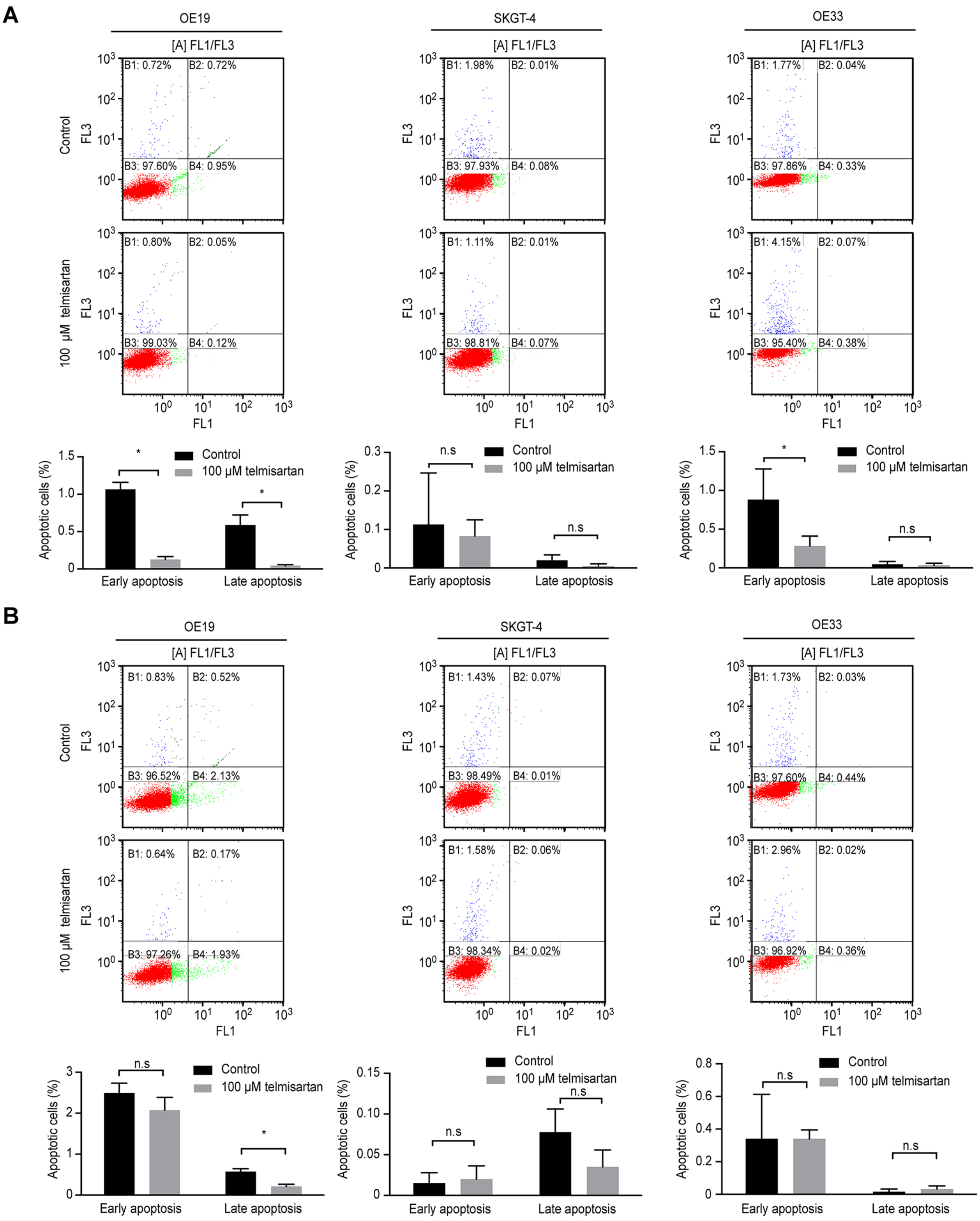

Supplementary Figure S2: Flow cytometry assessment of apoptosis of EAC cells (OE19, SKGT-4, OE33) with or without 100 µM telmisartan. A. At 24 h after telmisartan treatment. **B.** At 48 h after telmisartan treatment. n.s., not significant; *, $P<0.05$.

A

Reference Spots		Activin A	ADAMTS-1	Angiogenin	Angiopoietin-1	Angiopoietin -2	Angiostatin/ Plasminogen	Amphiregulin	Artemin		Reference Spots
Coagulation Factor 3	CXCL16	DPP 4	EGF	EG-VEGF	Endoglin	Endostatin Collagen18	Endothelin-1	FGF acidic	FGF basic	FGF-4	FGF-7
GDNF	GM-CSF	HB-EGF	HGF	IGFBP-1	IGFBP-2	IGFBP-3	IL-1β	IL-8	LAP(TGF- β1)	Leptin	MCP-1
MIP-1α	MMP-8	MMP-9	NRG1-β	Pentraxin 3 (PTX3)	PD-ECGF	PDGF-AA	PDGF-AB/ PDGF-BB	Persephin	Platelet Factor 4 (PF4)	PIGF	Prolactin
Serpin B5	Serpin E1	Serpin F1	TIMP-1	TIMP-4	Thrombospondin-1	Thrombospondin-2	uPA	Vasohibin	VEGF	VEGF-C	
Reference Spots											Negative Control

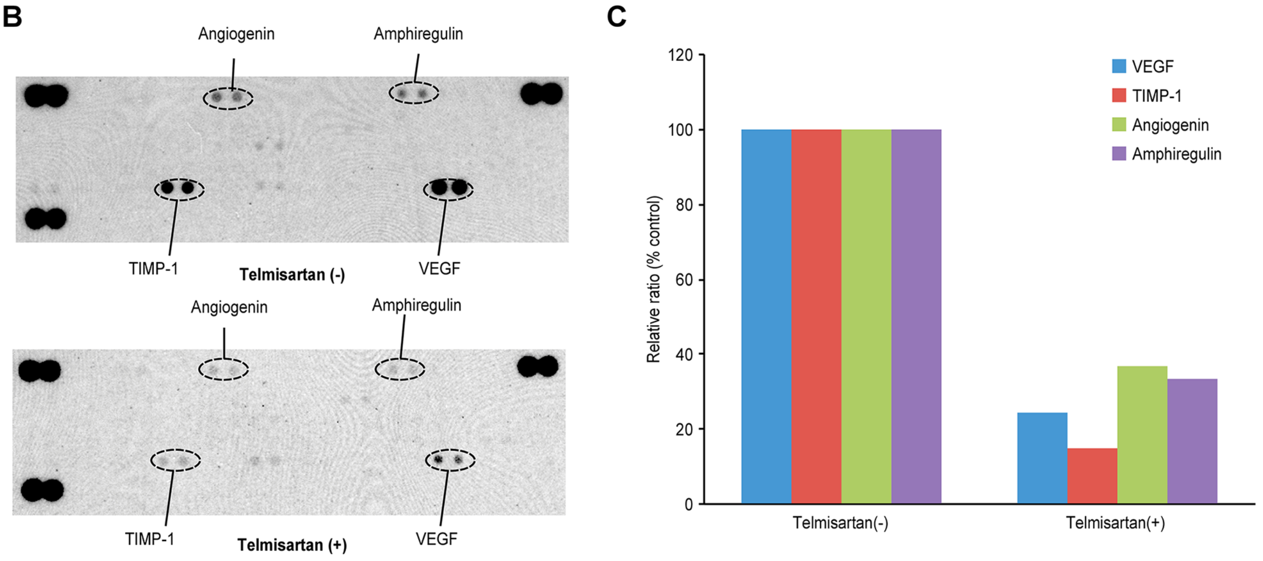

Supplementary Figure S3: Effects of telmisartan on angiogenesis in the OE19 cells. A. Template depicting the location of antibodies for angiogenesis-related proteins spotted onto a human angiogenesis array. **B.** Representative expression levels of various antibodies for angiogenesis-related proteins in the OE19 cells treated with or without telmisartan. Decreased expression levels of VEGF, TIMP-1, angiogenin, and amphiregulin were detected in the OE19 cells treated with telmisartan. **C.** Densitometry analysis indicated that the ratios of the VEGF, TIMP-1, angiogenin, and amphiregulin spots of the telmisartan-treated cells to those of the untreated cells were 24.3%, 14.9%, 36.9%, and 33.4%, respectively.

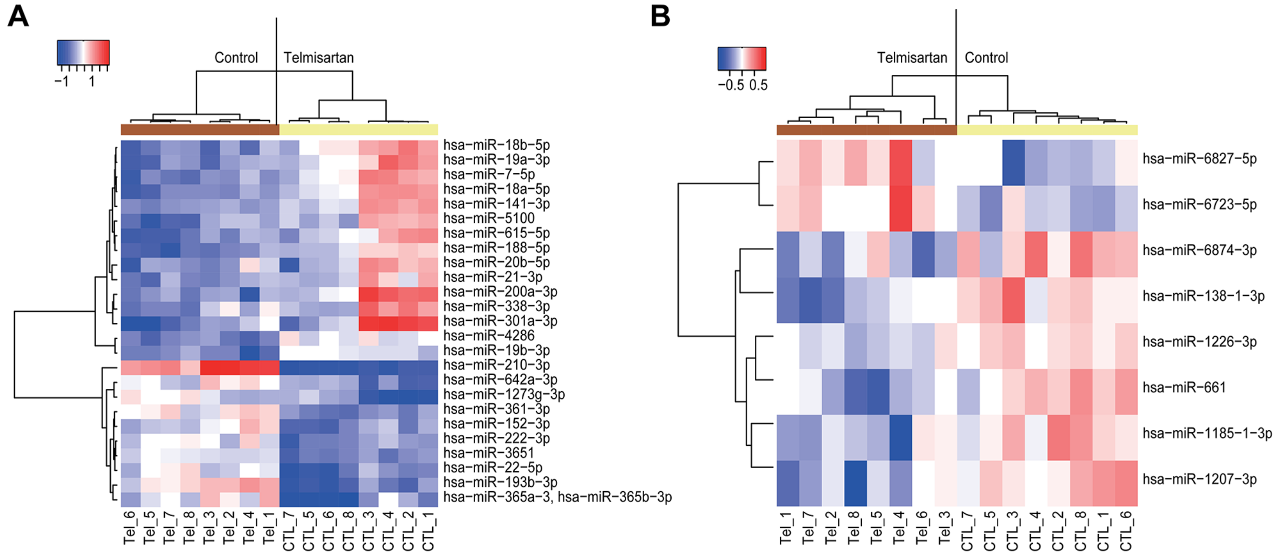

Supplementary Figure S4: A. Hierarchical clustering of OE19 cells treated with and without telmisartan. OE19 cells were clustered based on the expression profiles of 25 differentially expressed miRNAs between telmisartan-treated OE19 cells and untreated cells. **B.** Hierarchical clustering of tumor samples from xenograft animal models treated with telmisartan or vehicle. Tumor tissues were clustered based on the expression profiles of 8 miRNAs that were differentially expressed between tumor tissues from mice treated with telmisartan or vehicle. The analyzed samples are shown in the columns, and the miRNAs are presented in the rows. The miRNA clustering color scale shown at the top indicates the relative expression levels of the miRNAs, and red and blue represent high and low expression levels, respectively.

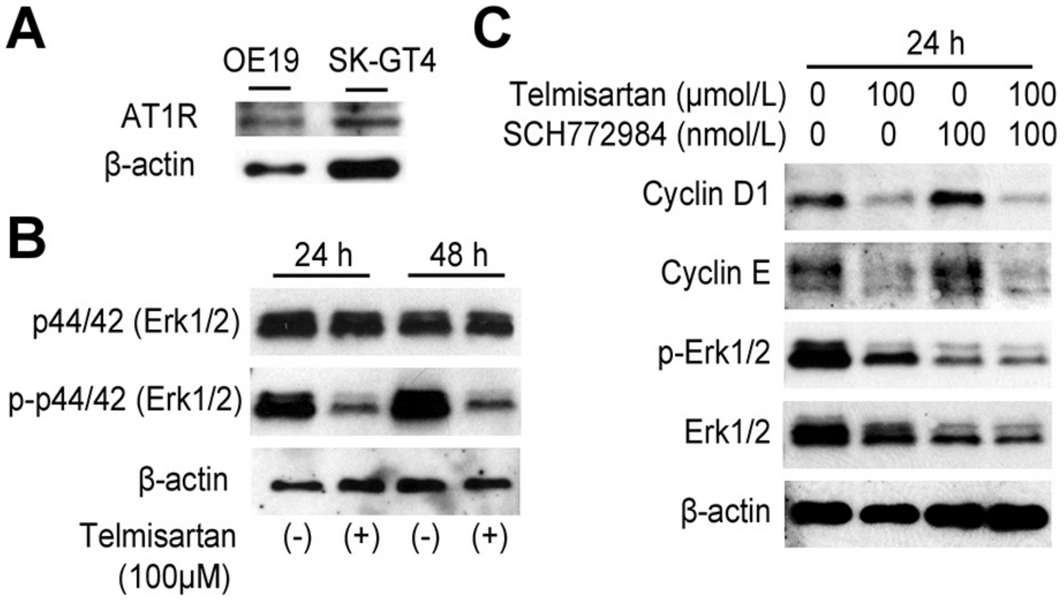

Supplementary Figure S5: Telmisartan does not induce cell cycle arrest via activation of the AT1R cascade. A. OE19 and SK-GT4 cells express the AT1 receptor. **B.** OE19 cells were treated with 100 μM telmisartan, and the activation status of the MEK/ERK pathway was determined. **C.** Western blot analysis of cyclin D1 and cyclin E in OE19 cells treated with control, telmisartan alone, SCH772984 alone, or telmisartan combined with SCH772984 for 48 h.

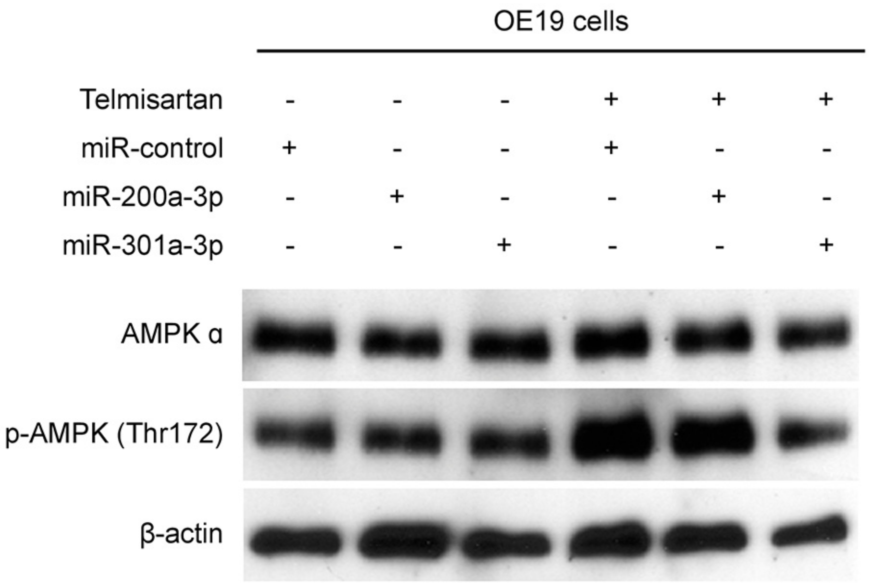

Supplementary Figure S6: The effect of miR-200a-3p and miR-301a-3p overexpression on the expression and phosphorylation of AMPKα in OE19 cells.

Supplementary Table S1: Statistical results and chromosomal locations of miRNAs in OE19 cells treated with and without telmisartan

miRNA	Fold change (Treated/Untreated)	*P*-value	Chromosomal location
**Up-regulated**			
hsa-miR-210-3p	5.53	<0.001	11p15.5
hsa-miR-193b-3p	2.41	<0.001	16p13.12
hsa-miR-365a-3p, hsa-miR-365b-3p	1.87	0.0074	16p13.12, 17q11.2
hsa-miR-361-3p	1.78	<0.001	Xq21.2
hsa-miR-22-5p	1.74	0.0019	17p13.3
hsa-miR-1273g-3p	1.67	0.0060	1
hsa-miR-642a-3p	1.65	0.0013	19q13.32
hsa-miR-222-3p	1.56	<0.001	Xp11.3
hsa-miR-152-3p	1.55	0.0074	17q21.32
hsa-miR-3651	1.52	<0.001	9
**Down-regulated**			
hsa-miR-301a-3p	0.36	0.0312	17q22
hsa-miR-200a-3p	0.41	0.0016	1p36.33
hsa-miR-7-5p	0.47	0.0013	9q21.32,15q26.1,19p13.3
hsa-miR-18b-5p	0.47	0.0019	Xq26.2
hsa-miR-18a-5p	0.47	<0.001	13q31.3
hsa-miR-19a-3p	0.48	0.0016	13q31.3
hsa-miR-615-5p	0.52	0.001	12q13.13
hsa-miR-141-3p	0.54	0.000	12p13.31
hsa-miR-188-5p	0.55	0.000	Xp11.23
hsa-miR-5100	0.56	0.012	10
hsa-miR-338-3p	0.60	0.046	17q25.3
hsa-miR-4286	0.60	0.001	8
hsa-miR-19b-3p	0.62	0.001	13q31.3,Xq26.2
hsa-miR-21-3p	0.63	0.005	17q23.1
hsa-miR-20b-5p	0.64	0.038	Xq26.2

Fold change (FC)>1.5, FC<0.67, *P*-value<0.05.

Supplementary Table S2: Statistical results and chromosomal locations of miRNAs in esophageal cancer tumors treated with and without telmisartan

miRNA	Fold change (Treated/Untreated)	*P*-value	Chromosomal location
**Up-regulated**			
hsa-miR-6827-5p	1.31	0.0030	3
hsa-miR-6723-5p	1.30	0.0038	1
**Down-regulated**			
hsa-miR-6874-3p	0.72	0.0030	7
hsa-miR-1207-3p	0.73	0.0030	8
hsa-miR-138-1-3p	0.74	0.0011	3p21.32
hsa-miR-661	0.76	0.0047	8q24.3
hsa-miR-1185-1-3p	0.76	0.0047	14
hsa-miR-1226-3p	0.86	0.0019	3p21.31

P-value<0.005.
